# Epidemiology of Chikungunya Virus Infection in the Philippines

**DOI:** 10.3390/reports9040364

**Published:** 2026-10-20

**Authors:** Nestor S. Arce, Hisham A. Imad, Prakaykaew Charunwatthana

**Affiliations:** 1 College of Medicine, Jose Maria College Foundation, Inc., Davao City 8000, Philippines; nestor.arce@jmc.edu.ph; 2 Department of Clinical Tropical Medicine, Faculty of Tropical Medicine, Mahidol University, Bangkok 10400, Thailand; hishamahmed.ima@mahidol.ac.th; 3 Thai Travel Clinic, Hospital for Tropical Diseases, Faculty of Tropical Medicine, Mahidol University, Bangkok 10400, Thailand; 4 Mahidol Oxford Tropical Medicine Research Unit, Faculty of Tropical Medicine, Mahidol University, Bangkok 10400, Thailand

**Keywords:** chikungunya, Philippines, outbreaks

## Abstract

**Background:** Chikungunya virus (CHIKV) has circulated in the Philippines for more than six decades. We systematically reviewed the epidemiology of CHIKV in the Philippines, integrating evidence on transmission, seroprevalence, diagnostics and molecular lineages. **Methods:** We searched PubMed and Embase to March 2026 for original studies reporting incidence, prevalence, seroprevalence, outbreak characteristics, or surveillance findings in human populations in the Philippines. The gray literature from the Department of Health (DOH) Epidemiology Bureau’s national chikungunya surveillance (2018–March 2026) was included separately. Findings were synthesized narratively because of substantial heterogeneity. **Results:** Sixteen studies and one national surveillance dataset were included. National surveillance recorded 12,662 cases from 2018 to early 2026, including 722 laboratory-confirmed cases and three fatalities. The Asian genotype predominated during the 2011–2013 epidemic, while Indian Ocean lineage (IOL) viruses were detected in southern Mindanao. **Conclusions:** Transmission of CHIKV in the Philippines is mostly sporadic with periods of low-level transmission. Because the clinical features overlap with dengue, acute febrile illness surveillance should test for both viruses.

## 1. Introduction and Clinical Significance

An arthritogenic mosquito-borne alphavirus belonging to the *Togaviridae* family, CHIKV was first isolated during the 1952–1953 Newala epidemic in southern Tanzania [1]. The outbreak was characterized by high attack rates and severe arthralgia, and the virus was successfully recovered from both affected patients and *Aedes aegypti* mosquitoes, establishing its role as the causative agent and confirming mosquito-borne transmission [2].

However, historical evidence suggests that CHIKV caused outbreaks long before its formal identification. Affected individuals were recognized primarily by their clinical presentation, predominated by debilitating arthralgia. This gave rise to descriptive local terms such as “knuckle fever,” first reported in 1779 in present day Jakarta, reflecting the prominent involvement of peripheral small joints. Similar clinical patterns were later documented during the same year in Cairo (1779), as well as in later major urban epidemics across India, most notably in Calcutta in 1824 [3].

In the Philippines, evidence of CHIKV circulation dates to investigations of the epidemic hemorrhagic fevers conducted in the 1950s. During a major 1956 outbreak of hemorrhagic fever in Manila and surrounding Luzon, Dr. William M. Hammon and colleagues suspected chikungunya because some children suffered from unusually severe crippling joint pain [4]. However, the virus itself was not isolated until 1965 by Dr. L. E. Campos and his team [5]. This timeline confirmed earlier suspicions regarding the earlier co-circulation of these two pathogens; historical reviews by virologists like Scott B. Halstead verified that during this period, dengue viruses were responsible for severe hemorrhagic cases while chikungunya co-circulated as the cause of a clinically similar but milder febrile illness in the region [6]. Consistent with these early descriptions, a 1968 case series from the Philippines involving 41 serologically confirmed patients reported a predominantly mild febrile illness characterized by rash and arthralgia (66%), without severe complications [7]. Sporadic infections were later identified among US Peace Corps volunteers in 1985–1986, suggesting a low transmission setting in the Philippines [8].

We conducted a systematic review of human CHIKV studies in the Philippines to summarize the epidemiological patterns and outbreak dynamics that may inform public health policies and disease control strategies.

## 2. Methodology

This systematic literature review was conducted according to the Preferred Reporting Items for Systematic Reviews and Meta-Analyses (PRISMA) 2020 guidelines to address the following question: “What is the epidemiology of CHIKV infection among individuals residing in the Philippines, including incidence, prevalence, seroprevalence, outbreak characteristics, attack rates, and disease burden in both laboratory-confirmed and clinically suspected cases?” The study protocol was registered in the International Prospective Register of Systematic Reviews (PROSPERO) (protocol CRD42024544293).

### 2.1. Information Sources

We systematically searched PubMed and Embase covering the literature available up to 31 March 2026. Reference lists of included papers were hand-searched. Additionally, we included the gray literature from the Philippine Department of Health (DOH) Epidemiology Bureau, specifically the national chikungunya surveillance dataset (2018–March 2026) obtained directly from the DOH. The extract was provided as regional aggregate counts of notified cases, laboratory-confirmed cases and deaths. The Bureau did not release individual line lists, addresses, specimen dates or testing denominators, so the extract cannot support geocoding, or linkages to vector density maps. Entomology only and non-human studies were not sought as primary sources.

### 2.2. Search Strategy

**Figure 1:**
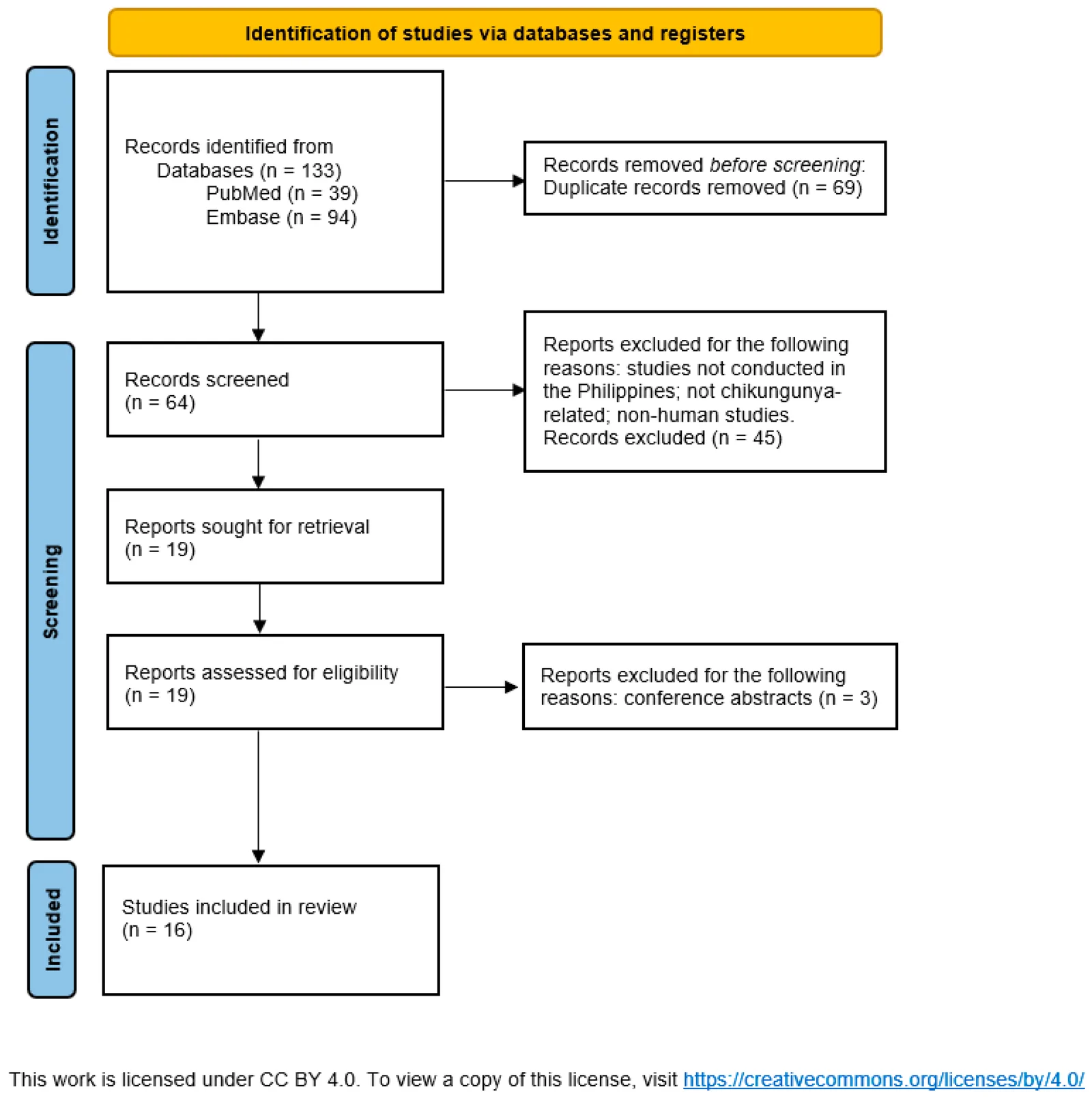
PRISMA 2020 flow diagram of search results from databases and registers. One additional gray-literature source (Philippine DOH national surveillance dataset, 2018–2026) was incorporated separately into the review [23].

The PubMed strategy combined title/abstract keywords and MeSH terms as follows: (chikungunya[Title/Abstract] OR CHIKV[Title/Abstract] OR “chikungunya virus”[MeSH] OR “chikungunya fever”[Title/Abstract] OR CHIK[Title/Abstract]) AND (Philippine*[Title/Abstract] OR Philippines[Title/Abstract] OR Manila[Title/Abstract] OR Cebu[Title/Abstract] OR Davao[Title/Abstract] OR Laguna[Title/Abstract] OR Mindanao[Title/Abstract] OR Luzon[Title/Abstract] OR Quezon[Title/Abstract] OR “Metro Manila”[Title/Abstract] OR Calabarzon[Title/Abstract] OR Visayas[Title/Abstract]) AND (epidemiolog*[Title/Abstract] OR outbreak*[Title/Abstract] OR incidence[Title/Abstract] OR prevalence[Title/Abstract] OR seroprevalen*[Title/Abstract] OR surveillan*[Title/Abstract] OR burden[Title/Abstract] OR “attack rate”[Title/Abstract] OR coinfect*[Title/Abstract] OR transmission[Title/Abstract] OR seroepidemiolog*[Title/Abstract] OR “disease burden”[Title/Abstract]). An equivalent Embase search used the same keywords mapped to Emtree (chikungunya, chikungunya virus, chikungunya fever; Philippines and named regions; epidemiology/outbreak/incidence/prevalence/surveillance). No date restriction was applied at search; language was restricted to English at full-text eligibility. The exact database strings, run dates, hit counts and PRISMA 2020 flow are given in Supplementary Table S2 and Figure 1. We did not search Scopus, Web of Science or local-language repositories.

### 2.3. Eligibility Criteria

Studies were eligible for inclusion if they were conducted in human populations in the Philippines and reported original epidemiological data or secondary analyses of surveillance datasets on CHIKV infection, including incidence, prevalence, seroprevalence, outbreak characteristics, surveillance findings, or disease burden. Eligible study designs included cross-sectional studies, cohort studies, case series, surveillance reports, outbreak investigations, and conference proceedings with epidemiological elements, provided they contributed unique human data not captured in full publications.

### 2.4. Selection Process

Two independent reviewers (N.S.A. and H.A.I.) screened titles and abstracts based on the eligibility criteria using Rayyan software version 1.7.8, classifying studies as included, excluded, or uncertain. Studies with uncertain relevance were retained to ensure that all potentially eligible articles were considered. Full-text articles were subsequently retrieved and independently assessed for eligibility. Disagreements were resolved through discussion, with a third reviewer (P.C.) involved when necessary. The same process was applied during full-text screening.

Only articles published in English were included. Studies were excluded if they were reviews, editorials, commentaries, or opinion pieces; if they reported only entomological or laboratory-based findings without human epidemiological outcomes; or if they were not specific to the Philippines. Multi-country studies were excluded unless Philippines-specific data could be extracted.

### 2.5. Data Collection Process

Two independent reviewers (N.S.A. and H.A.I.) extracted data using a standardized form. A second reviewer independently verified the extracted data to ensure completeness and accuracy. Any discrepancies were resolved through discussion, with a third reviewer (P.C.) involved if necessary.

### 2.6. Data Items

The data extraction form captured key study characteristics, including author, publication year, study design, location, population, sample size, diagnostic methods, and epidemiological outcomes such as incidence, prevalence, seroprevalence, outbreak characteristics, attack rates, and disease burden, as well as the main findings. When more than one article reported the same cohort, incident infections were extracted once. Those papers were treated as complementary analyses of a single population rather than as independent studies.

### 2.7. Synthesis of Results

Quantitative meta-analysis and meta-regression were not performed. The 16 studies and the DOH extract do not estimate a single commensurate parameter: they combine historical virus isolation, site-specific plaque-reduction neutralization tests, immunoglobulin M (IgM) among dengue-suspect inpatients, real time–polymerase chain reaction (RT-PCR) among outbreak sera, attack rate, and person-year incidence. A pooled prevalence or a single standardized incidence would not be interpretable across these designs.

### 2.8. Risk of Bias

All 16 articles were independently appraised with the Joanna Briggs Institute checklist that matched each study’s primary design. Items were scored yes, no, unclear or not applicable. We did not compute a single numeric score. Separately, we assigned an overall judgment of low, moderate or high concern for inference to CHIKV epidemiology in the resident Philippine population. Gray-literature DOH surveillance was described but not scored. No study was excluded on quality grounds. Ratings inform the narrative synthesis and are reported in full in Supplementary Table S1.

## 3. Results

A total of 133 records were identified through database searching (PubMed: 39; Embase: 94). After removal of duplicates and screening of titles and abstracts, 19 unique records were selected for full-text assessment. Three conference abstracts were excluded because they did not report unique Philippine epidemiological data beyond the full papers already included. Sixteen studies met the inclusion criteria and were included in the systematic review (Figure 1). One gray-literature source, the Philippines DOH national surveillance extract for 2018–March 2026, was incorporated separately and was not counted among the 16 studies.

### 3.1. Study Characteristics

The included studies comprised cross-sectional studies, prospective cohort studies, case series, and outbreak investigations, and one multi-country retrospective seroprevalence study from which Philippines-specific data were extracted. Settings included Cebu, Manila, Laguna, Mindanao, and other regions (Table 1). Study periods ran from the 1950s to 2020 in the published literature, with passive surveillance extended to March 2026. Sample sizes ranged from outbreak investigations and traveler case series to community cohorts and hospital serosurveys. Assays included IgM and immunoglobulin G enzyme-linked immunosorbent assay (IgG ELISA), plaque or focus-reduction neutralization (PRNT/FRNT), and RT-PCR.

Concern for inference to community epidemiology differed by sampling frame. The four Cebu papers describe one household cohort and were rated low for incidence and neutralizing immunity; incident infections should be counted once (n = 129: 25 symptomatic and 104 subclinical) [9,10,11,12]. A low-to-moderate rating was assigned for active pediatric surveillance, because of IgM only testing and a vaccine-site cohort [13]. A moderate rating was given for village census, with unmatched controls, and IgM testing on 58 of 84 sera [14]. A moderate rating was given for occurrence and low rating for lineage assignment [15,16]. Hospital and residual-sera series were rated moderate-to-high for community prevalence because the sampling frame was care-seeking febrile illness [17,18,19,20]. These studies do not estimate community incidence [5,21,22].

**Table 1:** Included studies of CHIKV infection in the Philippines (*n* = 16).

**A. Historical isolation and serosurveys**				
Campos 1969 [5]	Philippines, mid-1960s	Not reported	Mouse inoculation	First Philippine isolates
Tesh 1975 [24]	Luzon, Marinduque, Cebu, Mindanao; 1968–74	Residents, *n* = 284	PRNT	59/284 (14–30% by site)
Ngwe 2016 [18]	Dengue-suspect sera, 2001	*n* = 160	IgG ELISA + FRNT	IgG 57.5%; FRNT 43/160 (26.8%)
**B. Cebu prospective cohort**				
Yoon 2015 [9]	Cebu City, 2012–13	Community cohort, *n* = 853	PCR + PRNT	12.32 infections/100 PY; 19% symptomatic
Srikiatkhachorn 2016 [10]	Same cohort, 2012–14	*n* = 854–1008	PCR + PRNT	Year 1: 107 infections; year2: 21; NAb 28% to 42%
Yoon 2020 [11]	Same cohort, 2012–14	854 enrolled; 765 with paired titers	PRNT_80_	129 incident (25 symptomatic + 104 subclinical)
Salje 2016 [12]	Cebu, reconstructed 1952–2012	Model on 150 historical + 853 cohort sera	PRNT + catalytic model	Baseline 241/853 (28%); 0/294 aged <14 y
**C. 2011–2013 epidemic**				
Sy 2016 [15]	Nationwide; first detected in Mindanao, 2011–13	Surveillance sera, *n* = 5729	IgM ± PCR; E1 sequencing	2891 IgM-positive; 131 PCR-positive among IgM-negative
Tan 2015 [16]	Multi-province, 2012	19 isolates	Genome sequencing	18 Asian; 1 ECSA-IOL
Ballera 2015 [14]	Concepcion village, San Pablo, Laguna, 2012	7881 residents; 84 sera	Clinical definition + IgM	Attack rate 98/7881 (1.2%); 58/84 (69%) IgM-positive
Velasco 2015 [19]	Quezon City hospital, 2012–13	Dengue-like illness, *n* = 112	RT-PCR	11/112 (10%) CHIKV; 0 dengue coinfection
Nakayama 2018 [21]	Japan; infections acquired in the Philippines, 2012–13	3 travelers	RT-PCR + sequencing	3/3 Asian genotype
**D. Other hospital and pediatric studies**				
Inoue 2000 [17]	San Lazaro and St. Luke’s, Metro Manila, 1999	Dengue-suspect inpatients (PCR *n* = 315; IgM-IFA *n* = 590)	RT-PCR + multi-assay serology	PCR 0/315; IgM-IFA 90/590 (15.3%); IgM ELISA 47/343 (13.7%)
Capeding 2013 [13]	San Pablo and Cebu, children 2–14 y, 2010–11	Active fever cohort, *n* = 300	IgM	35 events; 21.3/100 PY (95% CI 15.3–29.6)
Arce 2024 [20]	Four provincial hospitals, Davao de Oro, 2019–20	Febrile inpatients, *n* = 382	IgM (± dengue markers)	55/382 (14.4%) any CHIKV IgM; 7.07% CHIKV only
**E. Ecological analysis of official reports**				
Cueva 2018 [22]	NEC regions, 2012–July 2013	Official confirmed reports	NEC case classification	777 + 562 regional counts

Abbreviations: ELISA, enzyme-linked immunosorbent assay; FRNT, focus-reduction neutralization test; IFA, immunofluorescence assay; IgG/IgM, immunoglobulin G/M; NAb, neutralizing antibody; NEC, National Epidemiology Center; PCR/RT-PCR, (reverse-transcription) polymerase chain reaction; PRNT/PRNT_80_, (80%) plaque-reduction neutralization test; PY, person-years; RR, relative risk.

### 3.2. Epidemiological Findings

The included studies were not pooled. The comparison was organized by timing of transmission, spatial clustering, lineage geography, seroprevalence and incidence, diagnostic methods, and circulating viral lineages.

#### 3.2.1. Timing of Transmission

CHIKV was suspected during investigation of epidemic hemorrhagic fever in 1956 and isolated in the mid-1960s [4,5]. By the late 1960s to the mid-1970s, neutralizing antibody was present on several islands. In the Tesh survey, the site-specific PRNT results were 13/58 adults sampled in 1968–1970 (22%), 8/58 in Tagudin, Luzon (14%), 18/101 in Marinduque (18%), and 20/67 in Atlas, Cebu (30%), totaling 59/284 [24]. Age-specific seroprevalence rose with age in Cebu and Mindanao. Later reports described sporadic infections among Peace Corps volunteers in 1986 and a community outbreak in Cavite in 1996.

Reconstructed waves in Cebu were concentrated in 1968–1969 (about 18% of remaining susceptibles; about 135,000 infections), 1986 (37%; about 190,000), and 1993 (14%; about 32,000), with a mean interval of 17 years between outbreaks [12]. The same model estimated that more than half of the Cebu City population remained susceptible after every outbreak and at every time point from 1952 to 2012. At enrolment in 2012, 241 of 853 participants (28%) were PRNT-positive, and none of 294 children younger than 14 years had detectable antibody [12].

In the 2012–2014 prospective Cebu cohort, incidence in the first year was 12.32 infections per 100 person-years overall and 16.9 per 100 person-years among participants seronegative for neutralizing antibody. In the second year, incidence fell to 2.84 per 100 person-years and seroprevalence rose from 28% to 42% [10].

#### 3.2.2. Spatial Clustering

Seroconversion and clinical illness were spatially clustered. Participants who seroconverted in 2012–2013 were 2.2 times as likely to live within 50 m of another seroconverter (95% CI 1.5–3.1) and 1.7 times as likely to live within 100 m (95% CI 1.2–2.3); clustering remained significant out to 230 m [12]. In Concepcion village, San Pablo City Laguna, 98 clinically defined cases occurred between 23 June and 6 August 2012 with an estimated attack rate of 1.2% (98/7881). Fifty-eight of 84 tested sera (69%) were CHIKV IgM-positive. In an unmatched case–control analysis, illness was associated with a chikungunya case in the household (aOR 6.2, 95% CI 3.0–12.9) and haphazard garbage disposal (aOR 2.7, 95% CI 1.4–5.4). House and Breteau indices were 27% and 28, with larvae predominantly *Aedes aegypti* [14].

**Figure 2:**
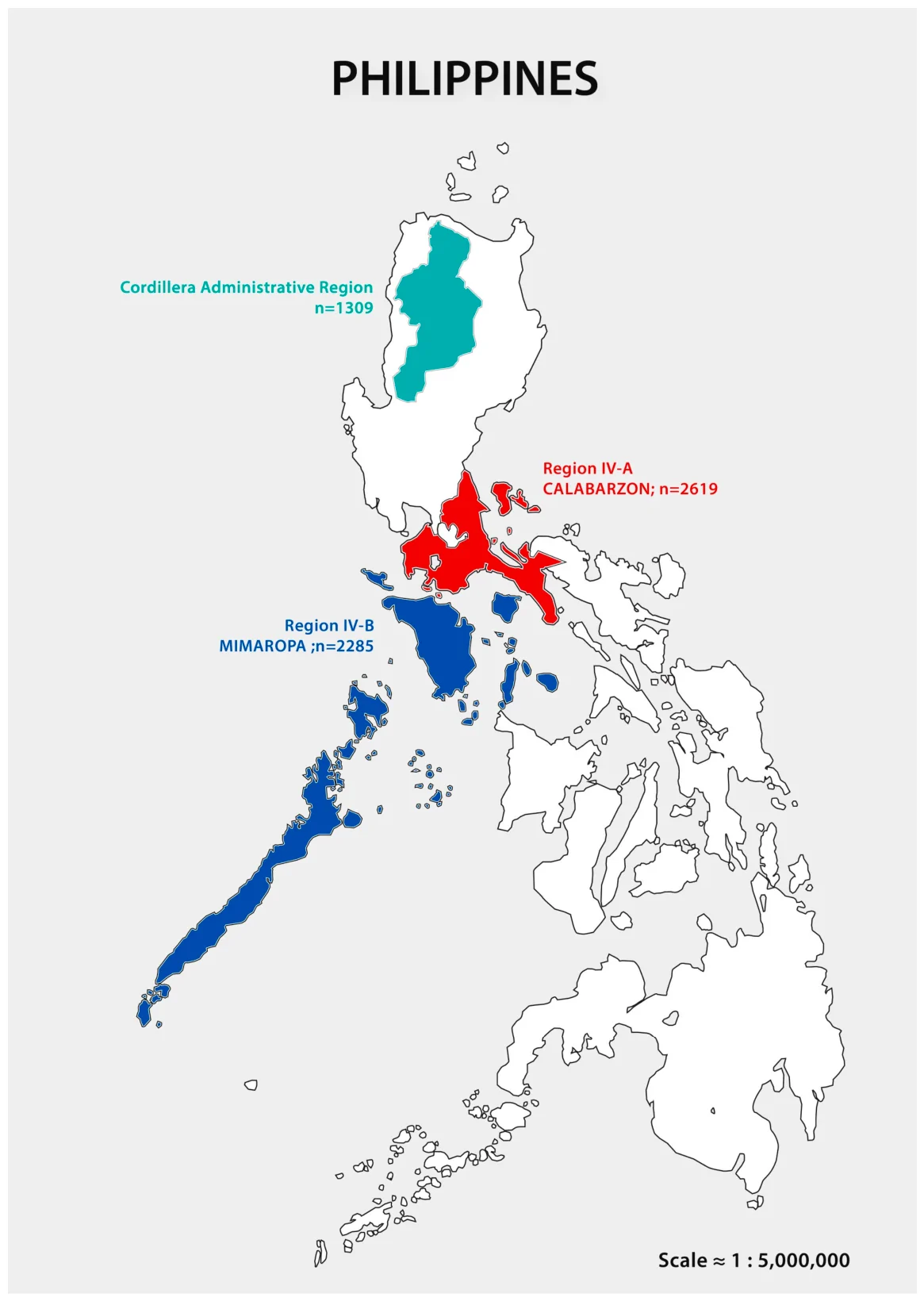
Notified chikungunya cases by region, Philippines, 2018–March 2026, from Department of Health passive surveillance.

Notified CHIKV cases in 2018–2026 were concentrated in CALABARZON, MIMAROPA, and CAR (Figure 2).

The DOH recorded 12,662 notified cases, of which 722 (5.7%) were laboratory-confirmed, and three deaths. High-resolution coordinates of the laboratory-confirmed cases were not available in the DOH extract.

All age groups were affected. Among notified cases, adults aged 25–34 years accounted for the largest share, followed by children aged 5–14 years and adults aged 60 years and older. In Davao de Oro in 2019–2020, CHIKV IgM-positive febrile patients were unevenly distributed across four provincial hospitals, with higher proportions at Laak and Pantukan than at Montevista [20].

An ecological analysis confirmed reports showed a rise in nationwide prevalence but no significant association with regional rainfall or urbanization levels [22].

#### 3.2.3. Lineage Geography

The 2011–2013 epidemic was first detected in Mindanao before being recognized elsewhere in the Philippines [15]. Two sequencing sets describe the geographic split. In the national surveillance set, partial E1 sequences were obtained from 31 viruses: 28 belonged to the Asian genotype and 3 to the East, Central and South African (ECSA) genotype. All three ECSA viruses were IOL and were found only in Davao del Sur and Davao Oriental in 2012–2013 [15]. Asian genotype viruses were identified from other parts of the country in the same period [15]. A second set of 19 isolates, chosen to represent different provinces, gave the same geographic pattern at lower resolution: 18 were of the Asian genotype and 1, CK12-455, was ECSA–IOL from Davao [16]. Phylogenetic analysis of the Asian genotype viruses favored an introduction from Indonesia. Full-genome analysis placed acquisition of nsP3-D483N in Compostela Valley, with subsequent E1-P397L and E3-Q19R [16]. Traveler and hospital sequences from Manila and from infections acquired in the Philippines were of the Asian genotype [19,21].

#### 3.2.4. Seroprevalence, Incidence and Notified Cases

In Cebu in 2012, baseline neutralizing antibody seroprevalence was 28% [9,11]. In the first year, subclinical incidence (10.03 per 100 person-years) exceeded symptomatic incidence (2.19 per 100 person-years); 19% of infections were symptomatic [9]. No symptomatic infections occurred among participants with a baseline PRNT titer > 10 [9].

Across both years, 25 symptomatic infections and 104 subclinical seroconversions occurred in participants with no detectable pre-year neutralizing antibody. Among those with a pre-year PRNT_80_ titer ≥ 1:10 there were no symptomatic infections and one subclinical seroconversion [11].

Chikungunya markers were also detected among patients investigated for dengue. In Manila in 1999, RT-PCR was negative (0/315), while IgM reactivity was 13.7–15.3% depending on the assay [17]. In 2001 residual sera, immunoglobulin G (IgG) ELISA positivity was 57.5% and FRNT positivity was 26.8% [18]. In Quezon City in 2012–2013, 11 of 112 patients (10%) with a clinical diagnosis of dengue were CHIKV RT-PCR positive, with no dengue coinfection [19]. In Davao de Oro in 2019–2020, CHIKV IgM was detected in 55 of 382 febrile inpatients (14.4%) [20]. In children aged 2–14 years in San Pablo and Cebu in 2010–2011, IgM incidence was 21.3 per 100 person-years (95% CI 15.3–29.6) [13].

The DOH recorded 12,662 cases from 2018 to March 2026, of which 722 (5.7%) were laboratory-confirmed, with three deaths. Annual totals were 2903 in 2018, 1131 in 2019, 157 in 2020, 93 in 2021, 601 in 2022, 2955 in 2023, 3900 in 2024, and 852 in 2025; 70 cases were reported in January–March 2026.

#### 3.2.5. Diagnostic Methods Across Studies

Diagnostic methods differed across studies. The 1999 Metro Manila study combined RT-PCR after C6/36 inoculation with antigen ELISA on culture fluid, IgM-IFA, IgM-capture ELISA, and IgG ELISA, without PRNT or FRNT [17]. Early multi-island work used plaque-reduction neutralization [24]. Later hospital and outbreak investigations used IgM-capture ELISA, with RT-PCR restricted to IgM-negative acute samples. During 2011–2013, 2891 of 5729 outbreak-surveillance sera were IgM-positive. Among 382 IgM-negative specimens subsequently tested by RT-PCR, 131 were positive for CHIKV [15].

#### 3.2.6. Molecular Epidemiology

Asian genotype sequences from the 2011–2013 outbreak were closely related, with E1 nucleotide similarity above 99%, and clustered with Indonesian strains, consistent with introduction before 2010 [15]. Phylogeographic analysis favored an introduction from Indonesia [15]. Full-genome analysis of 2012 isolates placed acquisition of nsP3-D483N in Compostela Valley, followed by E1-P397L and E3-Q19R in viruses linked to Laguna; this order is consistent with export after those substitutions were acquired [16]. An isolate from a viremic traveler infected in the Philippines in 2012 belonged to the Asian genotype and clustered with contemporaneous Pacific and Caribbean strains [21]. Two E1 sequences from Manila patients in 2012–2013 also belonged to the Asian genotype and clustered with viruses later reported from Micronesia and the Caribbean [19]. The ECSA viruses were a separate introduction. All three viruses identified by Sy et al. carried E1-A226V, a substitution associated with enhanced transmission by *Aedes albopictus*, and were closely related to a 2010 Indonesian virus [15,25]. CK12-455, the single ECSA isolate in Tan et al., also fell within the IOL [16]. Neither analysis placed the main 2012 outbreak in the ECSA–IOL; both placed it in the Asian genotype, described by Tan et al. as a cosmopolitan Asian genotype [16].

## 4. Discussion

Chikungunya transmission in the Philippines is best understood as sporadic and focal with low-level transmission punctuated by periodic outbreaks over approximately seven decades, since clinical recognition in the mid-1950s. Suspected chikungunya was noted in Manila and surrounding Luzon in 1956, with virus isolation in the mid-1960s, further transmission in the late 1980s and 1990s, and a large nationwide outbreak in 2011–2013. As reconstructed for Cebu, these waves infected only a minority of remaining susceptibles and it is estimated that more than half of the city’s population remain susceptible [12]. In the same cohort, transmission fell as neutralizing antibody prevalence rose from 28% to 42% [10]. DOH surveillance data from 2018 to early 2026 showed a similar temporal pattern, with 2903 reported cases in 2018, 2955 in 2023 and 3900 in 2024, and lower counts in the intervening years. This pattern is consistent with outbreaks that do not exhaust population susceptibility.

Epidemiological and genetic evidence also places the Philippines in the regional transmission networks rather than under a single persistent endemic lineage. In 2011–2013 the Asian genotype predominated, while the ECSA IOL viruses were detected in southern Mindanao [15,16]. These viruses carried E1-A226V, a substitution associated with enhanced transmission by *Aedes albopictus* [25]. Molecular surveillance after 2013 remains limited, making it difficult to determine whether ECSA viruses remained geographically restricted to southern Mindanao or whether subsequent local evolution and lineage replacement occurred. The scarcity of contemporary whole-genome data is an important gap.

Mortality was uncommon in the included studies. The Cebu cohort and the Davao de Oro hospital series reported no deaths [9,10,20]. DOH surveillance recorded three deaths among 12,662 notified cases (0.02%). The extract does not state whether these deaths were laboratory-confirmed, so a case fatality ratio among the 722 confirmed cases cannot be calculated. Mortality is therefore unlikely to be the main public health burden. None of the included Philippine studies measured the frequency of chronic musculoskeletal symptoms. Persistent symptoms are a recognized consequence of chikungunya infection in other endemic regions [26]. In the Philippines, the more significant consequences are therefore likely to be prolonged musculoskeletal symptoms in a subset of symptomatic patients and diagnostic confusion with dengue, rather than mortality.

Three operational implications follow. First, repeated serological neutralization-based serosurveys in the same populations would measure susceptibility more directly than passive case counts. Second, acute febrile illness surveillance should test for CHIKV and dengue together where the syndromes overlap. Third, sequencing of a sample of confirmed cases would show whether the Indian Ocean lineage persisted, remained restricted to southern Mindanao, or was replaced after 2013.

This review has limitations. Restriction to the English language may have excluded relevant local-language studies. The search covered two bibliographic databases and available national surveillance data, and may have missed provincial outbreak reports. Heterogeneity in populations, designs, case definitions and assays limited comparison and precluded meta-analysis. Much of the strongest longitudinal evidence comes from Cebu and may not represent Mindanao or areas such as the Cordillera Administrative Region. Hospital series preferentially capture patients who seek care. Finally, few contemporary genomes limit inference or persistence, replacement and geographic distribution of lineages.

Within those limits, the evidence supports sporadic, focal transmission, with accumulation of susceptibles between outbreaks. That pattern has persisted for several decades, so further outbreaks are likely, although their timing, location, size and dominant lineage remain uncertain. Repeated serosurveillance, CHIKV testing within acute febrile illness surveillance, molecular monitoring and targeted vector control are the measures most directly supported by the gaps identified here.

## 5. Conclusions

CHIKV transmission in the Philippines has occurred as sporadic, focal outbreaks separated by long intervals in which susceptibility can accumulate. Routine surveillance underestimates burden because many infections are subclinical or mild, and only a small fraction of recent notified cases were laboratory-confirmed. The Asian genotype predominated in 2011–2013, with IOL detected in southern Mindanao. These findings support the need for combined CHIKV-dengue testing in acute febrile illness surveillance and for periodic serological and molecular monitoring.

## Data Availability

No new primary data; included studies and DOH surveillance as cited.

## Supplementary Materials

The following supporting information can be downloaded at: https://www.mdpi.com/article/10.3390/reports9040364/s1, Table S1. Risk of bias of included studies using design-matched JBI checklists; Table S2. Search strategy and reproducibility.

## Abbreviations

The following abbreviations are used in this manuscript:

AFI	acute febrile illness
aOR	adjusted odds ratio
CALABARZON	Cavite, Laguna, Batangas, Rizal, and Quezon
CAR	Cordillera Administrative Region
CFR	case fatality ratio
CHIK	chikungunya (clinical/shorthand usage)
CHIKV	chikungunya virus
CI	confidence interval
DF	dengue fever
DHF	dengue hemorrhagic fever
DOH	Department of Health (Philippines)
ECSA	East/Central/South African (genotype)
ELISA	enzyme-linked immunosorbent assay
FRNT	focus-reduction neutralization test
FRNT_80_	80% focus-reduction neutralization titer
IFA	immunofluorescence assay
IgG	immunoglobulin G
IgM	immunoglobulin M
IOL	Indian Ocean lineage
MeSH	Medical Subject Headings
MIMAROPA	Mindoro, Marinduque, Romblon, and Palawan
NAb	neutralizing antibody
NEC	National Epidemiology Center
PCR	polymerase chain reaction
PRISMA	Preferred Reporting Items for Systematic Reviews and Meta-Analyses
PRNT	plaque-reduction neutralization test
PRNT_80_	80% plaque-reduction neutralization titer
PROSPERO	International Prospective Register of Systematic Reviews
PY	person-years
RNA	ribonucleic acid
RR	relative risk
RT-PCR	reverse-transcription polymerase chain reaction
