# Supplementary material for: Epidemiology of Chikungunya Virus Infection in the Philippines
Source: Reports (MDPI). 2026 Oct 20;9(4):364. doi: 10.3390/reports9040364 (PMC13692436; doi:10.3390/reports9040364)
Supplement: Supplementary file 1 [file reports-09-00364-s001.zip › reports-4584857-supplementary.pdf]

Table S1. Risk of bias of included studies using design-matched JBI checklists

**Tesh 1975**	Prevalence	Partial (selected islands)	Unclear	Yes (PRNT)	Unclear	Yes	Moderate
**Ngwe Tun 2016**	Prevalence	No (residual dengue-suspect sera)	No	Yes (FRNT)	NA	Yes	High
**Yoon 2015**	Cohort	Yes (Cebu community)	Yes (random household)	Yes (PCR + PRNT)	Yes	Yes	Low
**Srikiatkhachorn 2016**	Cohort	Yes (same Cebu cohort)	Yes	Yes (PCR + PRNT)	Yes	Yes	Low
**Yoon 2020**	Cohort	Yes (same Cebu cohort)	Yes	Yes (PRNT80)	Yes	Yes	Low
**Salje 2016**	Prevalence + catalytic model	Yes (1973 + 2012 Cebu titres)	Yes	Yes (PRNT)	Yes	Yes	Low
**Sy 2016**	Prevalence	Partial (national suspects)	Unclear	Yes (IgM ± PCR + E1)	Unclear	Yes	Moderate
**Tan 2015**	Case series	No (available isolates)	No	Yes (whole genome)	NA	Yes	Moderate*
**Ballera 2015**	Case-control	Yes (village census)	Partial (unmatched controls)	Partial (clinical + IgM subset)	Yes	Yes	Moderate
**Velasco 2015**	Prevalence	No (tertiary dengue-like illness)	No	Yes (RT-PCR)	NA	Yes	High
**Nakayama 2018**	Case series	No (Japan travellers)	No	Yes (RT-PCR + sequencing)	NA	Yes	High†
**Inoue 2000**	Prevalence	No (dengue-suspect inpatients)	No	Partial (PCR negative; IgM discordant)	NA	Partial	High
**Capeding 2013**	Cohort	Partial (2 PH sites, ages 2–14 y)	Yes	Partial (IgM ELISA only)	Yes	Yes	Low–moderate
**Arce 2024**	Prevalence	No (4 provincial hospitals)	No	Partial (IgM ± dengue markers)	Yes	Yes	Moderate
**Campos 1969**	Case series	No	No	Yes (isolation)	NA	Unclear	High
**Cueva 2018**	Analytical cross-sectional	Official counts only	NA	No (no individual assay)	NA	Partial	High

**Tan 2015 is low concern for lineage description of the 19 sequenced 2012 isolates. †Nakayama 2018 is low concern for documenting export of the 2012 Asian lineage. DOH Epidemiology Bureau surveillance (2018–March 2026) is grey literature and was not scored with a JBI checklist. JBI tools: Prevalence Studies (Munn et al. 2015); Cohort Studies (Moola et al. in JBI Manual); Case Series (Munn et al.); Case Control Studies (Moola et al.); Analytical Cross-sectional Studies (Barker et al. 2026).*

Table S2. Search strategy and reproducibility

Item	Specification
Protocol	PROSPERO CRD42024544293
Databases	PubMed; Embase
Last search date	31 March 2026
PubMed hits	39
Embase hits	94
Records after screening / full text / included	19 full text assessed; 16 studies included; 3 conference abstracts excluded because later published in full
Grey literature	DOH Epidemiology Bureau national chikungunya extract, 1 Jan 2018–31 Mar 2026; regional aggregates of notified cases, laboratory-confirmed cases and deaths; no line list or geocode
PubMed string	(chikungunya[Title/Abstract] OR CHIKV[Title/Abstract] OR "chikungunya virus"[MeSH] OR "chikungunya fever"[Title/Abstract] OR CHIK[Title/Abstract]) AND (Philippine*[Title/Abstract] OR Philippines[Title/Abstract] OR Manila[Title/Abstract] OR Cebu[Title/Abstract] OR Davao[Title/Abstract] OR Laguna[Title/Abstract] OR Mindanao[Title/Abstract] OR Luzon[Title/Abstract] OR Quezon[Title/Abstract] OR "Metro Manila"[Title/Abstract] OR Calabarzon[Title/Abstract] OR Visayas[Title/Abstract]) AND (epidemiolog*[Title/Abstract] OR outbreak*[Title/Abstract] OR incidence[Title/Abstract] OR prevalence[Title/Abstract] OR seroprevalen*[Title/Abstract] OR surveillan*[Title/Abstract] OR burden[Title/Abstract] OR "attack rate"[Title/Abstract] OR coinfect*[Title/Abstract] OR transmission[Title/Abstract] OR seroepidemiolog*[Title/Abstract] OR "disease burden"[Title/Abstract])
Embase string	Equivalent keywords mapped to Emtree (chikungunya / chikungunya virus / chikungunya fever; Philippines and named island groups and cities;

	epidemiology OR outbreak OR incidence OR prevalence OR surveillance OR seroprevalence). Run on the same date.
Other sources	Reference lists of included papers; no Scopus, Web of Science or local-language repository search
Screening	Rayyan; two independent reviewers (N.A.J., H.A.I.); third reviewer P.C. for disagreements
Risk of bias	Design-matched JBI checklists; see Supplementary Table S1
